# *OsIAA18*, an Aux/IAA Transcription Factor Gene, Is Involved in Salt and Drought Tolerance in Rice

**DOI:** 10.3389/fpls.2021.738660

**Published:** 2021-11-18

**Authors:** Feibing Wang, Haofei Niu, Dongqing Xin, Yi Long, Guangpeng Wang, Zongmei Liu, Gang Li, Fan Zhang, Mingyang Qi, Yuxiu Ye, Zunxin Wang, Baolei Pei, Laibao Hu, Caiyong Yuan, Xinhong Chen

**Affiliations:** ^1^School of Life Sciences and Food Engineering, Huaiyin Institute of Technology, Huai’an, China; ^2^Huaiyin Institute of Agricultural Sciences of Xuhuai Region, Huai’an, China; ^3^Institute of Botany, Jiangsu Province and Chinese Academy of Sciences, Nanjing, China

**Keywords:** *OsIAA18*, rice, overexpression, salt and drought tolerance, ABA signaling

## Abstract

Auxin/indoleacetic acid (Aux/IAA) proteins play an important regulatory role in the developmental process of plants and their responses to stresses. A previous study has shown that constitutive expression of *OsIAA18*, an Aux/IAA transcription factor gene of rice improved salt and osmotic tolerance in transgenic *Arabidopsis* plants. However, little work is known about the regulatory functions of the *OsIAA18* gene in regulating the abiotic stress tolerance of rice. In this study, the *OsIAA18* gene was introduced into the rice cultivar, Zhonghua 11 and the *OsIAA18* overexpression in rice plants exhibited significantly enhanced salt and drought tolerance compared to the wild type (WT). Moreover, overexpression of *OsIAA18* in rice increased endogenous levels of abscisic acid (ABA) and the overexpression of *OsIAA18* in rice plants showed hypersensitivity to exogenous ABA treatment at both the germination and postgermination stages compared to WT. Overexpression of *OsIAA18* upregulated the genes involved in ABA biosynthesis and signaling pathways, proline biosynthesis pathway, and reactive oxygen species (ROS)-scavenging system in the overexpression of *OsIAA18* in rice plants under salt and drought stresses. Proline content, superoxide dismutase (SOD), and peroxidase (POD) activities were significantly increased, whereas malonaldehyde (MDA), hydrogen peroxide (H_2_O_2_), and superoxide anion radical (O_2_^–^) content were significantly decreased in the transgenic plants under salt and drought stresses. Taken together, we suggest that *OsIAA18* plays a positive role in drought and salt tolerance by regulating stress-induced ABA signaling. The *OsIAA18* gene has a potential application in genetically modified crops with enhanced tolerance to abiotic stresses.

## Introduction

Environmental abiotic stresses, such as salt and drought, impact the growth, development, and productivity of agricultural crops and are becoming serious threats to provide and satisfy the needs of a rapidly growing population worldwide (Munns and Tester, 2008; Wang et al., 2016; Zhu et al., 2020). Plant adaptation to salt and drought stresses is dependent on the activation of cascades of molecular networks involved in stress perception, signal transduction, and the expression of specific stress-related genes and metabolites (Huang et al., 2012; Zhang et al., 2014). Rice is one of the most important grain crops and its production has been significantly affected by salt and drought stresses (Joo et al., 2019). Rice establishes a complex defense network in the process of resisting and adapting to abiotic stresses. It is important to understand abiotic stress responses of rice for enhancing salt and drought tolerance.

Auxin plays a very important role in a wide variety of plant developmental and physiological processes (Friml, 2003; Song et al., 2009; Li G. et al., 2020; Li W. et al., 2020). Auxin signaling can regulate the expression of downstream genes and perform the required responses by recruiting specific transcription factors (Vogler and Kuhlemeier, 2003). Auxin/indoleacetic acid (Aux/IAA) and auxin response factor (ARF) family proteins, as two important protein families of plants, play important roles in the developmental process and responses to phytohormones and stress treatments by means of controlling auxin-responsive transcription (Hagen and Guilfoyle, 2002; Liscum and Reed, 2002; Berleth et al., 2004; Song et al., 2009; Song and Xu, 2013). Aux/IAA proteins are short-lived transcription factors that are characterized by the presence of four conserved domains, known as domains I, II, III, and IV. Domain I, which is similar to the conserved domain of ethylene response factors functioning as transcription repressors, can inactivate ARF function, thereby repressing auxin-responsive transcription (Hiratsu et al., 2003; Tiwari et al., 2003). Domain II has been shown to be essential for auxin signaling by interacting with a component of the ubiquitin-proteasome protein degradation pathway (Gray et al., 2001; Ramos et al., 2001; Song and Xu, 2013). Domains III and IV of the C-terminal dimerization mediate homodimerization and heterodimerization among Aux/IAA and ARF proteins (Ulmasov et al., 1997; Hardtke et al., 2004; Song and Xu, 2013).

In order to determine the molecular mechanism of auxin signaling, the genes encoding Aux/IAA proteins have been cloned and identified in mung bean (Yamamoto, 1994), *Arabidopsis* (Reed, 2001), rice (Thakur et al., 2001; Nakamura et al., 2006), and grapevine (Çakir et al., 2013; Li G. et al., 2020). Many members of the Aux/IAA gene families in a variety of plant species are induced in response to auxin (Guilfoyle and Hagen, 2001; Thakur et al., 2001, 2005; Kepinski and Leyser, 2004). It has been reported that thirty-one Aux/IAA (*OsIAA*) genes of rice were identified and their sequences were analyzed (Jain et al., 2006; Wang et al., 2007). The first rice *OsIAA* gene, named *OsIAA13*, was cloned in the original reports (Thakur et al., 2001, 2005), while the first Aux/IAA protein characterized in rice was OsIAA31 (Nakamura et al., 2006). The *OsIAA23* gene played an important role in the postembryonic maintenance of a quiescent center (Jun et al., 2011). *OsIAA13* was involved in lateral root initiation (Kitomi et al., 2012). *OsIAA11* affected lateral root development (Zhu et al., 2012). Song et al. (2009) found that most of the *OsIAA* genes were responsive to various abiotic stresses, indicating an interaction between plant growth and abiotic stresses. However, the functions of most *OsIAA* genes in rice remain to be elucidated.

These are few reports on the overexpression of the related *IAA* genes in plants. The *OsIAA31* overexpression in rice plants showed insensitivity to auxin and gravitropic stimuli and exhibited short leaf blades, reduced crown root formation, and abnormal leaf formation (Nakamura et al., 2006). Overexpression of *OsIAA4* in rice induced morphological changes and reduced responsiveness to auxin (Song and Xu, 2013). Overexpression of *OsIAA6*/*OsIAA20* enhanced the tolerance to salt and drought stress in rice (Jung et al., 2015; Zhang et al., 2021). The *VvIAA18* gene was cloned from grapevine and it was found that the *VvIAA18*-overexpressing tobacco plants exhibited improved salt tolerance (Li W. et al., 2020). The *OsIAA18* gene was induced by salt, drought, IAA, and abscisic acid (ABA) treatments in rice (Song et al., 2009). Li G. et al. (2020) reported that heterologous overexpression of *OsIAA18* enhanced the tolerance to salt and osmotic stresses in transgenic *Arabidopsis* plants. However, little work is known about the regulatory functions of the *OsIAA18* gene in regulating the abiotic stress tolerance of rice.

In this study, the *OsIAA18* gene was further introduced into rice to characterize its functions. The functional analysis of *OsIAA18* was determined by investigating ABA sensitivity and salt and drought tolerance of *OsIAA18* overexpression in rice plants. Our results indicate that the *OsIAA18* gene is a positive regulator in rice salt and drought stress responses by ABA signaling and has potential application in genetically modified crops with enhanced tolerance to abiotic stresses.

## Materials and Methods

### Plant Materials and Transformation of Rice With *OsIAA18*

Rice (*Oryza sativa* L.) plants were grown in an experimental field at the Huaiyin Institute of Technology under natural conditions. Zhonghua 11 was used as the wild type (WT). EHA105, the *Agrobacterium tumefaciens* strain harboring a binary vector, plasmid pCAMBIA1301, was used in this study (Li G. et al., 2020). This binary vector contains the *OsIAA18* gene under the control of CaMV 35S promoter and nopaline synthase terminator (NOS) terminator of the expression box. This vector also contained *gusA* and *hpt*IIgenes driven by a CaMV 35S promoter. Then the recombinant vector was transformed into Zhonghua11 *via* the *A*. *tumefaciens*-mediated method (Hiei et al., 1994). After transformation, the callus was selected from 1/2 Murashige and Skoog (MS) medium containing 100 μg/mL hygromycin. Seedlings with hygromycin resistance were transplanted to soil in a growth chamber.

### DNA Extraction and PCR Detection

The DNA was extracted from rice leaves according to the instructions of EasyPure Plant Genomic DNA Kit (Transgen, Beijing, China). The presence of the *OsIAA18* expression construct in positive plants was assessed by PCR analysis using specific primers (Supplementary Table 1). PCR amplifications were performed with an initial denaturation at 94°C for 3 min, followed by 35 cycles at 94°C for 30 s, 55°C for 30 s, 72°C for 1 min, and a final extension at 72°C for 10 min. PCR products were separated by electrophoresis on a 1.0% (w/v) agarose gel.

### Salt and Drought Tolerance Assays

According to the methods of Li et al. (2017), the seeds of overexpressed *OsIAA18* and WT lines were germinated on 1/2 medium under 16-h light (28°C)/8-h dark (25°C) photoperiod conditions for 1 week and transplanted to 1/2 MS medium with no stress, 200 mM of NaCl, and 200 mM of mannitol, respectively. Plant length and fresh weight of *OsIAA18* overexpression and WT lines were measured after 1 week. The 4-week-old seedlings of *OsIAA18* overexpressed rice and WT lines were grown in 9-cm diameter pots containing a mixture of soil and vermiculite (1:1, v/v) in a greenhouse. All pots were irrigated sufficiently with water for 2 weeks under optimum growth conditions. Each pot was then irrigated with 100 m of 200 mM NaCl solution once every 2 days for 2 weeks, or subjected to drought stress for 1 week by stopping irrigation and recovery with rewatering for 1 week. Seedlings were regarded as survivals if the fresh and green young leaves emerged after water supply. The survival rates of these plants were observed immediately. All treatments were performed in triplicate.

With respect to the salt and drought stresses, the assays of the isolated leaves, leaf sections of approximately 5 cm in length from overexpressed *OsIAA18* rice and WT lines at seedling stages were removed and immersed in MS liquid media solutions with concentrations of 200 mM of NaCl and 200 mM of mannitol for 96 h, respectively. Disks that floated on sterile distilled water served as an experimental control. All the leaves were subsequently cultured under 16-h light (28°C)/8-h dark (25°C) photoperiod conditions. Each treatment was repeated three times, and 10 leaves were selected for each treatment. The total chlorophyll content of the rice leaves was measured according to the methods of Patra et al. (2010).

### RNA Extraction, Complementary DNA Synthesis, and Real-Time Quantitative Reverse Transcription -PCR Assay

Total leaf RNA was extracted from the transgenic plants and WT using the RNAprep Pure Plant Kit (Tiangen Biotech, Beijing, China). RNA samples were reverse-transcribed using Quantscript Reverse Transcriptase Kit (Tiangen Biotech, Beijing, China). The complementary DNA (cDNA) solution was used as a template for PCR amplification with specific primers (Supplementary Table 1). The expression of genes related to IAA signaling pathway, ABA biosynthesis, and signaling pathways, proline biosynthesis, stress responses, and ROS scavenging in the leaves of transgenic rice plants and WT grown in pots and incubated for 1 week under optimum growth condition, for 1 week under 200 mM of NaCl stress or for 1 week under drought stress were analyzed by real-time quantitative reverse transcription (qRT)-PCR as described by Wang et al. (2016). The *OsActin* gene of rice was used as an internal control, and it was amplified by specific primers (Supplementary Table 1). Quantification of the gene expression was done with the comparative *C*_T_ method (Schmittgen and Livak, 2008). All treatments were performed in triplicate.

### Abscisic Acid Content and Sensitivity Assays

Endogenous ABA levels in the leaves of transgenic rice plants and WT grown in pots and incubated for 1 week under optimum growth condition, for 1 week under 200 mM of NaCl stress or for 1 week under drought stress were performed by indirect ELISA as described by Wang et al. (2016). The ABA sensitivity at germination and postgermination stages was determined according to the methods of Li et al. (2017). The seeds of the overexpressed *OsIAA18* and WT lines were germinated on 1/2 MS medium with 2.5 μM of ABA, and the germination rates of the treated seeds were calculated after 5 days. The seeds of overexpressed *OsIAA18* and WT lines germinated on 1/2 MS medium with no stress were transplanted to 1/2 MS medium with 2.5μM of ABA; then, the shoot and root lengths and fresh weight of all lines were measured after 1 week. All treatments were performed in triplicate.

### 3,3′-Diaminobenzidine and Nitro Blue Tetrazolium Staining

The 3,3′-diaminobenzidine (DAB) staining and nitro blue tetrazolium (NBT) staining for hydrogen peroxide (H_2_O_2_) and superoxide anion radical (O_2_^–^) detection in the leaves of transgenic rice plants and WT grown in pots and incubated for 1 week under optimum growth condition, for 1 week under 200 mM of NaCl stress or for 1 week under drought stress, respectively, were performed as described by Jiang et al. (2016). All treatments were performed in triplicate.

### Measurements of IAA, Proline, H_2_O_2_, and Malondialdehyde Content

The IAA content in the leaves of transgenic rice plants and WT grown in pots and incubated for 1 week under normal growth conditions was analyzed based on the methods of Liu et al. (2012). The levels of proline, H_2_O_2_, and malondialdehyde (MDA) in the leaves of transgenic rice plants and WT grown in pots and incubated for 1 week under optimum growth condition, for 1 week under 200 mM of NaCl stress or for 1 week under drought stress, respectively, were measured as described (Wang et al., 2016). All treatments were performed in triplicate.

### Analyses of 9-*Cis*-Epoxycarotenoid Dioxygenase, Superoxide Dismutase, and Peroxidase Activities

The 9-cis-epoxycarotenoid dioxygenase (NCED) activity in the leaves of transgenic rice plants and WT grown in pots and incubated for 1 week under optimum growth condition, for 1 week under 200 mM of NaCl stress or for 1 week under drought stress, was measured with Plant NCED ELISA Kit (Uscn Life Science Inc., Shanghai, China). The activities of superoxide dismutase (SOD) and peroxidase (POD) were measured according to the method of Wang et al. (2016) and Li G. et al. (2020), respectively. All treatments were performed in triplicate.

### Statistical Analysis

The experiments were repeated three times and the data were presented as the mean ± standard error (SE). Wherever applicable, the data were analyzed by Student’s *t*-test in a two-tailed analysis. Values of *P* < 0.05 or < 0.01 were considered to be statistically significant.

## Results

### Overexpression of *OsIAA18* in Transgenic Rice Lines

**FIGURE 1:**
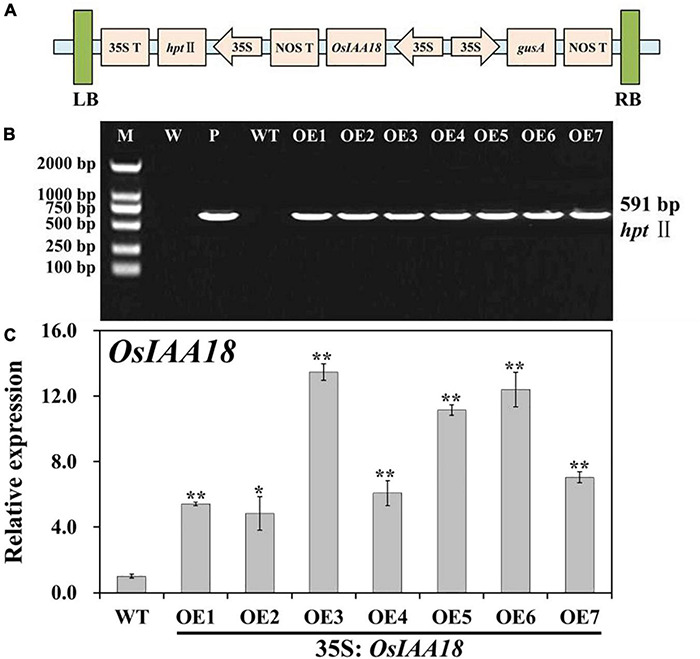
Molecular analyses of the *OsIAA18*-overexpressing rice plants. **(A)** Schematic diagram of the T-DNA region of binary plasmid pCAMBIA1301-*OsIAA18*. LB left border; RB right border; *hpt*II hygromycin phosphotransferase II gene; *OsIAA18* rice auxin/indoleacetic acid (Aux/IAA) transcription factor gene; *gusA* β-glucuronidase gene; 35S cauliflower mosaic virus (CaMV) 35S promoter; 35S T CaMV 35S terminator; NOS T nopaline synthase terminator. **(B)** PCR analysis of transgenic rice plants. Lane M, DL2000 DNA marker; Lane W, water as negative control; Lane P, plasmid pCAMBIA1301-*OsIAA18* as positive control; Lane WT, wild type; Lanes OE1, OE2, OE3, OE4, OE5, OE6, and OE7, *overexpressing OsIAA18* rice plants. **(C)** Expression level of *OsIAA18* in the overexpressing *OsIAA18* rice plants. The rice *OsActin* gene was used as an internal control. The results are expressed as relative values based on WT as reference sample set to 1.0. Data are presented as means ± SE (*n* = 3). The values for * and ** indicate a significant difference from that of WT at *P* < 0.05 and < 0.01, respectively, by Student’s *t*-test.

In our previous study, we confirmed that constitutive expression of *OsIAA18* significantly enhanced tolerance to salt and osmotic stresses in *Arabidopsis* plants (Li G. et al., 2020). To explore whether *OsIAA18* plays an important role in improving the agronomic traits through gene manipulation approaches, we introduced this gene to rice (*Oryza sativa L.* ssp. japonica cv. Zhonghua 11). The ORF of *OsIAA18* was overexpressed in rice cv. Zhonghua 11 (WT) using the binary vector pCAMBIA1301-*OsIAA18* (Figure 1A). Multiple lines were obtained from Hyg resistance selection. Seven independent transgenic lines of overexpressing *OsIAA18* gene in rice (T_1_ generation), named OE1, OE2, OE3, OE4, OE5, OE6, and OE7 were obtained, and their progenies (T_3_ generation) were generated. PCR analysis of genomic DNA indicated that they were transgenic (Figure 1B). These transgenic plants were further confirmed to have higher expression of the *OsIAA18* gene by qRT-PCR (Figure 1C), especially OE3, OE5, and OE6 plants. Therefore, the transgenic lines OE3, OE5, and OE6 were selected for further experiments.

**FIGURE 2:**
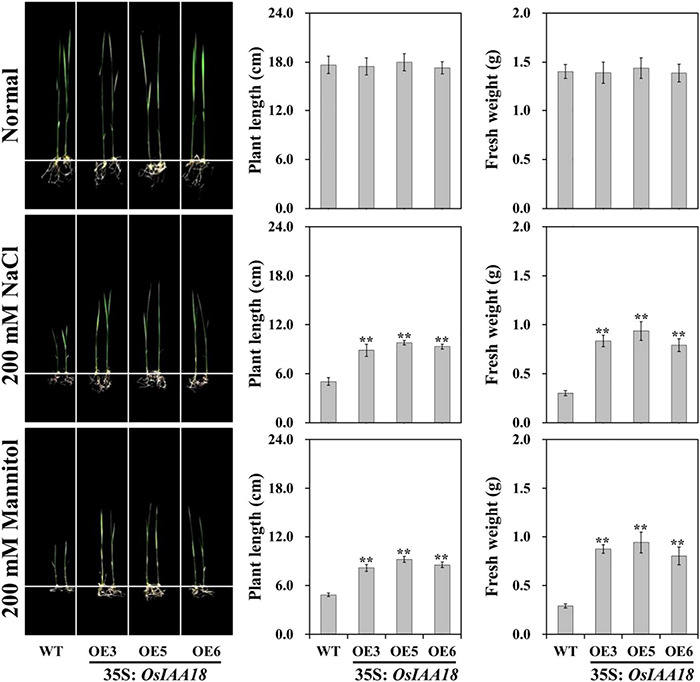
Growth performance of overexpressing *OsIAA18* and WT seedlings under 200 mM of NaCl and 200 mM of mannitol, respectively. Plant length and fresh weight of 7th-day old seedlings after transplantation. Data are presented as means ± SE (*n* = 3). The values of * and ** indicate a significant difference from that of WT at *P* < 0.05 and < 0.01, respectively, by Student’s *t*-test.

**FIGURE 3:**
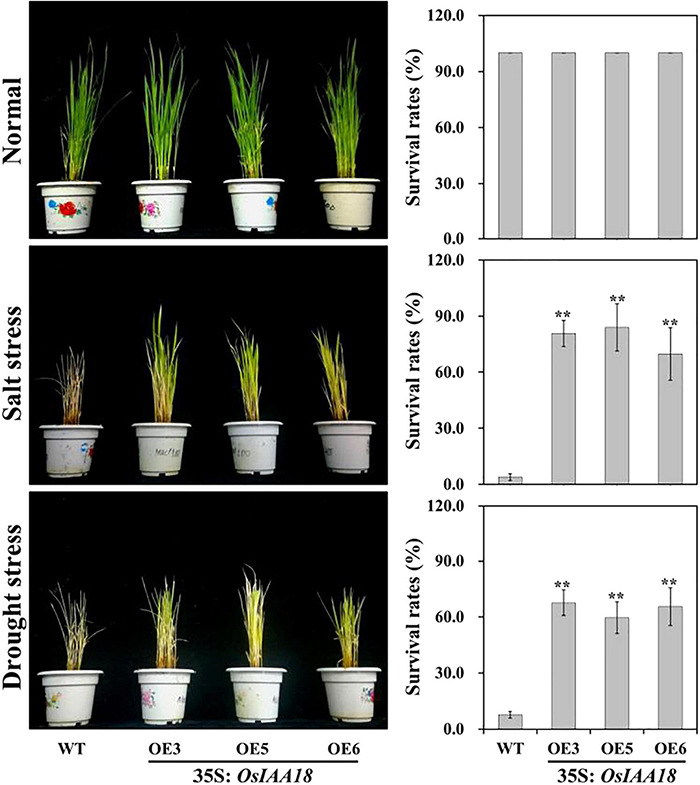
Responses of the transgenic rice plants and WT grown in pots under salt and drought stresses. Transgenic plants and WT were grown in pots and incubated for 2 weeks under normal conditions, for 2 weeks under 200 mM NaCl stress, and for 2 weeks by stopping irrigation and recovery with rewatering for 2 weeks. Survival rates of overexpressing OsIAA18 and WT plants after salt and drought stresses were examined. Data are presented as means ± SE (*n* = 3). The values of * and ** indicate a significant difference from that of WT at *P* < 0.05 and < 0.01, respectively, by Student’s *t*-test.

Under normal conditions, there were no significant differences in plant height and root length, and fresh weight between the overexpressing and WT lines grown at seedling stages (Figure 2). At the tillering stages, there were no developmental differences between *OsIAA18* overexpression and WT plants at normal irrigation (Figure 3). Furthermore, endogenous IAA levels and the expression levels of the genes related to IAA signaling pathway were analyzed in the leaves of overexpressed *OsIAA18* and WT plants under control growth conditions. The results showed that no obvious difference of IAA content was found under normal conditions (Supplementary Figure 1). There were no significant differences between *overexpressing OsIAA18* and WT plants in the expression levels of IAA biosynthesis-related YUCCA-like family gene *OsYUCCA6* and IAA responsive genes, *OsIAA6* and *OsIAA8* (Supplementary Figure 1).

### Improved Salt and Drought Tolerance of Overexpressing *OsIAA18* in Rice

Three lines (OE3, the OE5, and OE6) with the highest transcription levels of *OsIAA18* were selected to verify the functions of *OsIAA18* gene to the responses of stress treatments. The performance of overexpressing *OsIAA18* and WT lines under osmotic stress by adding 200 mM of NaCl or 200 mannitol was examined, respectively. It was worth noting that no developmental differences were identified between the overexpressing and WT lines under controlled growth conditions (Figure 2). Under salt and mannitol stresses, the growth of the overexpressing *OsIAA18* seedlings was less inhibited, which exhibited higher plant length and fresh weight than those of the WT seedlings (Figure 2). Together, these results suggest that the overexpression of *OsIAA18* in rice could enhance the tolerance to salt and mannitol stresses at seedling stages.

To investigate the functions of *OsIAA18* in response to salt and drought stresses, the overexpressing *OsIAA18* and WT seedlings were grown in the soil and were well-watered at the tillering stages. There were no developmental differences between overexpressing *OsIAA18* and WT plants when normal irrigation was performed (Figure 3). After 2 weeks of 200 mM NaCl stress, or 2 week after the stopping of irrigation and 2 weeks of recovery, the overexpressing *OsIAA18* seedlings remained green and showed continuous growth, whereas WT seedlings showed severe leaf rolling and wilting (Figure 3). This study also determined the survival rate for overexpressing *OsIAA18* and WT seedlings grown in the soil with salt and drought stresses. As shown in Figure 3, the survival rates of overexpressing *OsIAA18* lines were significantly higher than that of WT seedlings when exposed to salt and drought stresses. Therefore, it is evident that the overexpression of *OsIAA18* results in enhanced tolerance to salt and drought stresses in rice.

**FIGURE 4:**
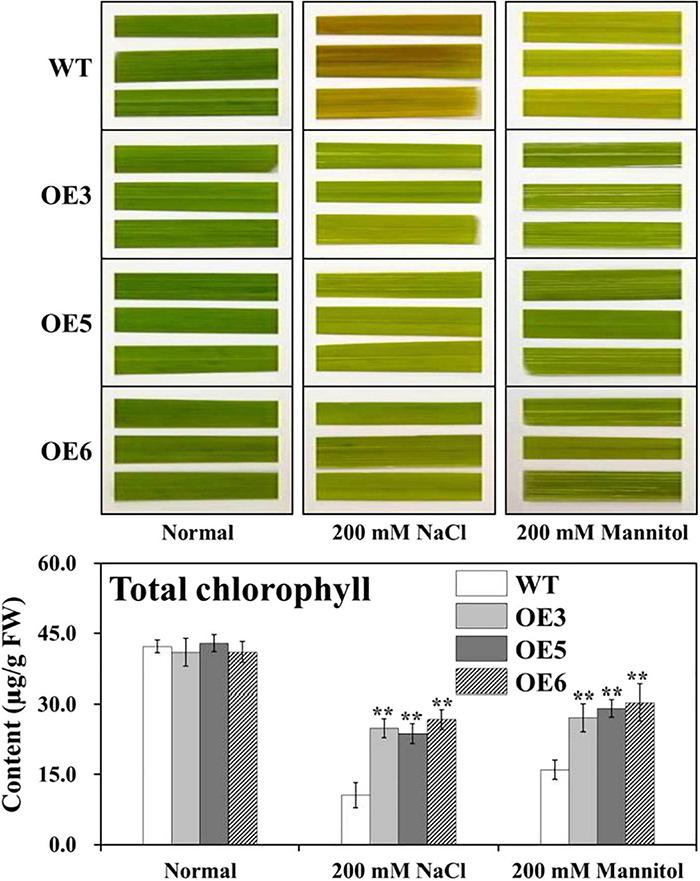
Assessment of the NaCl- and mannitol-induced senescence in leaf disks of various transgenic plants. Leaf disks were floated on 200 mM of NaCl and 200 mM of mannitol concentrations, respectively, for 96 h at 28°C under conditions of 28°C and a 16 h light/8 h dark photoperiod cycle. Disks floated on sterile distilled water served as an experimental control. Determination of total chlorophyll content of the leaf disks after NaCl and Mannitol treatments, respectively, are presented. Data are presented as means ± SE (*n* = 3). The values of * and ** indicate a significant difference from that of WT at *P* < 0.05 and < 0.01, respectively, by Student’s *t*-test.

As an explanation for the growth performance exhibited by the different transgenic plants, the possibility of a correlation between growth performance and the amount of chlorophyll retained in these plants during growth under salt and drought stresses was looked for. For such experiments, uniformly cut leaf disks from overexpressing *OsIAA18* and WT plants were incubated in the presence of an indicated concentration of 200 mM of NaCl or 200 mM of mannitol for 96 h at 28°C, respectively, and the “greenness” of the plants was compared against the control set without any stress (Figure 4). Although not very well discernible visually, the actual determination of chlorophyll content in the leaf disks showed that the overexpressing *OsIAA18* rice plants exhibited higher total chlorophyll content compared to WT (Figure 4), indicating that the transgenic plants were capable of retaining the chlorophyll to a greater extent in comparison to WT during growth under stress treatments. These results indicate that the overexpression of *OsIAA18* in rice increases salt and drought tolerance.

### Increased Sensitivity to Exogenous ABA in the Overexpression of *OsIAA18* in Rice

**FIGURE 5:**
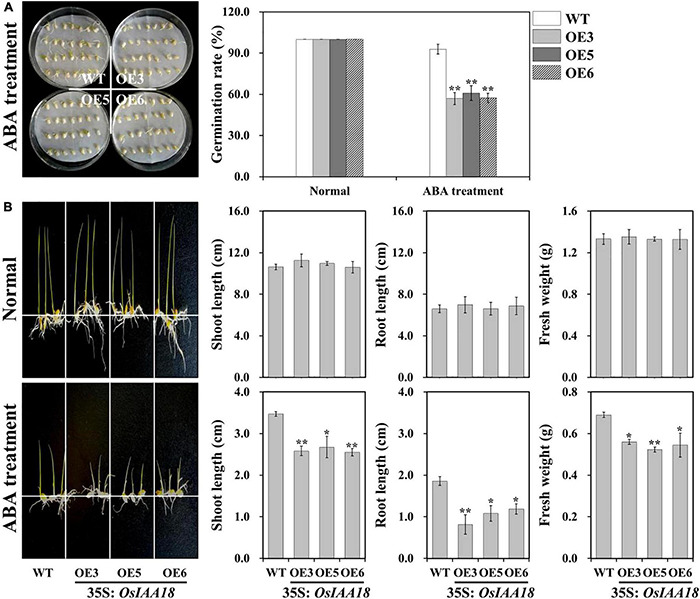
Increased ABA sensitivity of overexpressing *OsIAA18* rice plants during germination and seedling stages. **(A)** Germination assay of *OsIAA18* overexpression and WT seeds in 1/2 MS medium with 2.5 μM ABA. The germination rates of the treated seeds were calculated after 5 days. **(B)** Growth performance of overexpressing *OsIAA18* and WT plants grown in 1/2 MS medium containing 0 and 2.5μM ABA. Shoot and root lengths and fresh weight measurements of the rice plants are shown in panel. Data are presented as means ± SE (*n* = 3). The values of * and ** indicate a significant difference from that of WT at *P* < 0.05 and < 0.01, respectively, by Student’s *t*-test.

To test whether the overexpression of the *OsIAA18* gene can affect the sensitivity of transgenic rice to exogenous ABA, the overexpressing *OsIAA18* and WT seeds were germinated in 1/2 MS medium with ABA (0 and 2.5 μM). As shown in Figure 5A, the germination rate of the overexpressing *OsIAA18* lines was similar to that of WT control at 0 μM ABA. However, the germination rate of the overexpressing *OsIAA18* lines was significantly decreased at 2.5 μM ABA. These results suggested that the ABA sensitivity of seed germination of overexpressing *OsIAA18* rice plants was increased.

In our later study, we tested the effect of ABA on seedling development in overexpressing *OsIAA18* plants. The performance of overexpressing *OsIAA18* and WT seedlings grown for 2 weeks in 1/2 MS medium with or without ABA was observed (Figure 5B). There were no significant differences in shoot and root lengths and fresh weight between the WT and transgenic rice plants grown in MS medium without ABA (Figure 5B). The shoot and root lengths and the fresh weight of overexpressing *OsIAA18* plants grown in MS medium with 2.5 μM of ABA were significantly reduced compared to those of the control (Figure 5B). These results demonstrate that the overexpression of *OsIAA18* makes transgenic seedlings hypersensitive to ABA in comparison to WT plants, indicating that *OsIAA18* may be a positive regulator of ABA signaling in rice.

### Enhanced Endogenous ABA Signaling in Overexpressed *OsIAA18* Rice

**FIGURE 6:**
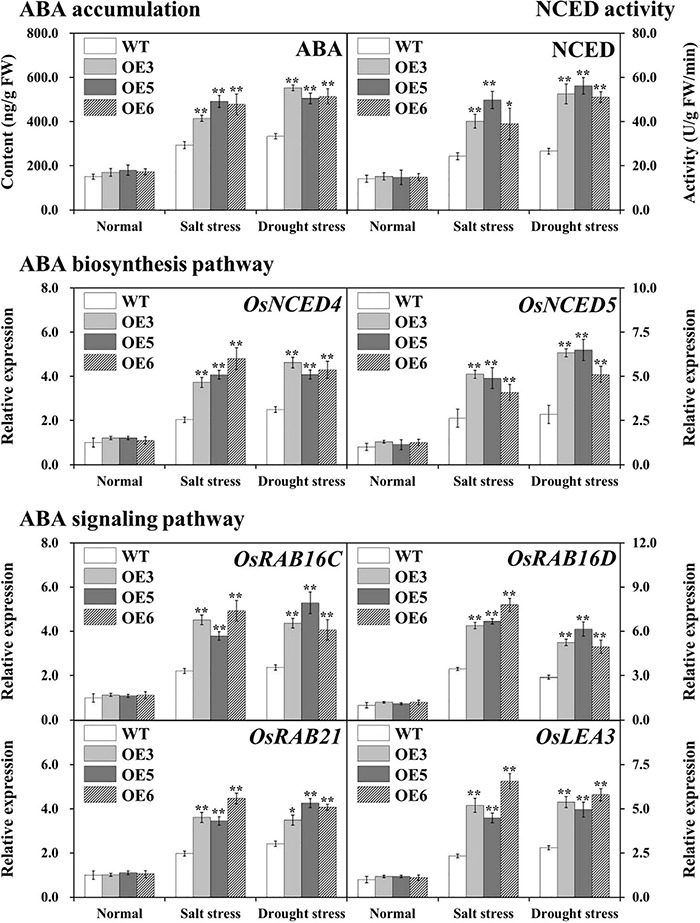
ABA accumulation and the expression of ABA biosynthesis and signaling pathways marker genes in the leaves of overexpressing *OsIAA18* and WT plants under salt and drought stresses. Data are presented as means ± SE (*n* = 3). The values of * and ** indicate a significant difference from that of WT at *P* < 0.05 and < 0.01, respectively, by Student’s *t*-test.

Endogenous ABA levels were measured in the leaves of overexpressing *OsIAA18* and WT plants under salt and drought stresses. The results showed that after salt and drought stresses, endogenous ABA levels were clearly increased in both overexpressing *OsIAA18* and WT lines; the accumulation of high ABA levels was observed in the overexpressing *OsIAA18* lines than that in the WT lines, while no obvious difference was found under normal conditions (Figure 6). It is hypothesized that *OsIAA18* gene may be involved in regulating salt and drought tolerance by ABA biosynthesis. To further confirm this hypothesis, the activity of 9-cis-epoxycarotenoid dioxygenase (NCED), as a key rate limiting enzyme of ABA biosynthesis, and the transcript levels of *OsNCED4* and *OsNCED5* were analyzed in overexpressing *OsIAA18* and WT plants under salt and drought stresses. As shown in Figure 6, the activity of NCED and the expression of *OsNCED4* and *OsNCED5* were enhanced in overexpressing *OsIAA18* lines compared to WT lines. Further research found that the transcript levels of ABA-responsive signaling pathway genes, such as *OsRAB16C*, *OsRAB16D*, *OsRAB21*, and *OsLEA3*, were markedly upregulated in the overexpressing *OsIAA18* lines compared to WT lines under salt and drought stresses (Figure 6). These results indicate that *OsIAA18* gene may play an important role in regulating salt and drought tolerance by increasing the expression of ABA biosynthesis genes, such as *OsNCED4* and *OsNCED5*, and upregulating the expression of *OsRAB16C*, *OsRAB16D*, *OsRAB21*, and *OsLEA3* genes related to ABA responsive signaling pathway.

### Promoted Proline Accumulation in Overexpressing *OsIAA18* Rice

**FIGURE 7:**
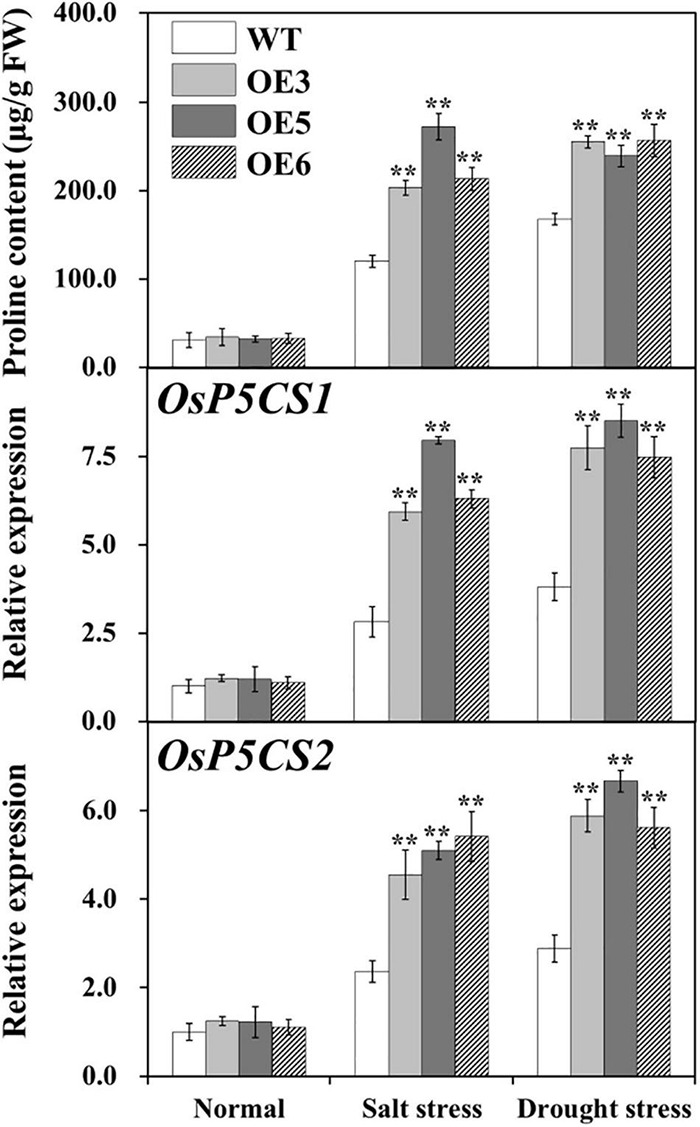
Free proline content and the expression of proline biosynthesis genes in the leaves of overexpressing *OsIAA18* and WT plants under salt and drought stresses. Data are presented as means ± SE (*n* = 3). The values for * and ** indicate a significant difference from that of WT at *P* < 0.05 and < 0.01, respectively, by Student’s *t*-test.

Proline content was analyzed in the leaves of overexpressing *OsIAA18* and WT plants under salt and drought stresses. These results showed that proline accumulation was much more significant in the overexpressing *OsIAA18* lines than those of the WT seedlings under salt and drought stresses (Figure 7). Meanwhile, we performed qRT-PCR assays to further analyze the messenger RNA (mRNA) levels of key rate-limiting enzyme genes related to proline biosynthesis under salt and drought stresses. Our data indicated that the expression of *OsP5CS1* and *OsP5CS2*, encoding the pyrroline-5-carboxylate synthase was upregulated in the overexpressing *OsIAA18* lines than those of the WT seedlings under salt and drought stresses (Figure 7). These results indicate that *OsIAA18* may play a critical role in stress-induced proline biosynthesis by the upregulation of proline biosynthesis genes.

### Decreased Reactive Oxygen Species Damage in Overexpressing *OsIAA18* Rice

**FIGURE 8:**
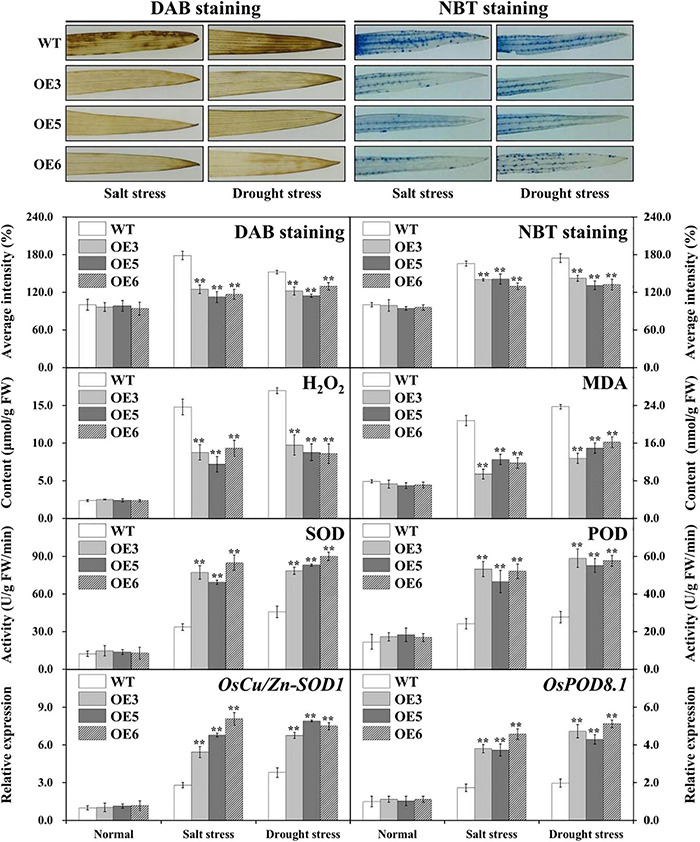
Decreased ROS damage in the overexpressing *OsIAA18* and WT plants. DAB and NBT staining for H_2_O_2_ and O_2_^–^, H_2_O_2_ and MDA content, SOD and POD activities, expression of *OsCu/Zn-SOD1* and *OsPOD8.1* genes in the leaves of transgenic plants and WT under salt and drought stresses. Data are presented as means ± SE (*n* = 3). The values of * and ** indicate a significant difference from that of WT at *P* < 0.05 and < 0.01, respectively, by Student’s *t*-test.

Stress usually induces damage *via* oxidative damage in plants including the generation of ROS, represented H_2_O_2_ and O_2_^–^ (Jiang et al., 2016). The accumulated levels of H_2_O_2_ and O_2_^–^ were evaluated by means of DAB and NBT staining and H_2_O_2_ measurements in the leaves of overexpressed *OsIAA18* and WT plants under salt and drought stresses. Our works showed that the overexpressed *OsIAA18* lines accumulated less H_2_O_2_ and O^2–^ than did WT lines under salt and drought stresses, whereas there was no significant difference between the transgenic plants and WT without stress (Figure 8). Meanwhile, we found that the overexpressed *OsIAA18* lines had significantly lower MDA than those of the WT lines (Figure 8).

Following salt and drought stresses, the activities of antioxidant enzymes, such as SOD and POD, that play an important role in ROS-scavenging mechanisms, were measured in both the overexpressing *OsIAA18* and WT lines. Under the absence of stress, SOD and POD activities were not different; after salt and drought stresses, the activities of these enzymes were significantly enhanced in the overexpressing *OsIAA18* lines compared to those in the WT lines (Figure 8). A qRT-PCR was used to further analyze the expression levels of *OsCu/Zn-SOD1* and *OsPOD8* in rice plants. Under salt and drought stresses, *OsCu/Zn-SOD1* and *OsPOD8* exhibited markedly higher expression in the overexpressing *OsIAA18* lines than in the WT lines (Figure 8). These results suggested that the overexpression of *OsIAA18* gene may enhance ROS-scavenging ability to decrease ROS damage, leading to improved salt and drought tolerance of transgenic rice lines.

## Discussion

### *OsIAA18* Plays a Positive Role in Enhancing Salt and Drought Tolerance in Rice

The Aux/IAA protein of rice plays an important regulatory role in the developmental process of plants and their responses to stress and phytohormone treatments (Song et al., 2009; Song and Xu, 2013).

The *OsIAA18* gene was upregulated when exposed to salt and drought stresses in rice (Song et al., 2009). Our previous study showed that the *OsIAA18* gene was isolated from a rice cultivar, Nipponbare and the *OsIAA18*-overexpressing *Arabidopsis* plants exhibited enhanced salt and osmotic tolerance (Li G. et al., 2020). Also, *VvIAA18* gene from the grapevine was cloned from PN40024 and it was found that the *VvIAA18*-overexpressing tobacco plants showed improved salt tolerance (Li W. et al., 2020). The *OsIAA6*/*OsIAA20*-overexpressing rice plants exhibited greater tolerance to salt and drought stresses (Jung et al., 2015; Zhang et al., 2021). In this study, we produced the transgenic plants of the rice cv. Zhonghua 11 overexpressing the *OsIAA18* gene. Under normal conditions, there were no significant differences in the plant height and root length, and fresh weight and tillers were observed at the tillering stages of the rice seedlings between the overexpressing *OsIAA18* and WT lines. Similar to the results reported by Jung et al. (2015) and Zhang et al. (2021), in which the overexpression of *OsIAA*6/*OsIAA20* had no significant effects on performance, such as plant height, root length, and tiller number, under normal conditions. Under salt and drought stresses, the overexpression of *OsIAA18* can significantly enhance the tolerance to these stresses in rice (Figures 2, 3). It is thought that the *OsIAA18* gene plays an important role in response to salt and drought stresses of rice and can be directly used in the genetic engineering of crops for their tolerance to abiotic stresses.

### *OsIAA18* Confers Salt and Drought Tolerance Through an ABA-Dependent Pathway in Rice

**FIGURE 9:**
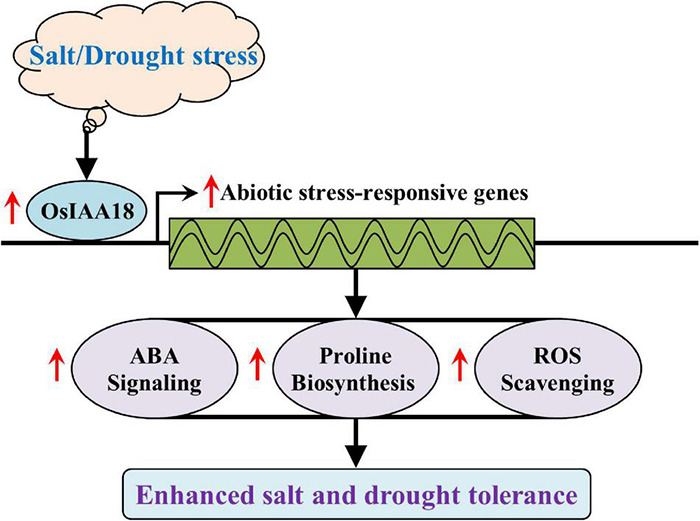
A proposed model explaining the function of *OsIAA18* in response to salt and drought stresses. Under salt and drought stresses, the overexpression of *OsIAA18* upregulated the expression of abiotic stress-responsive genes involved in ABA signaling, proline biosynthesis, and ROS scavenging, leading to increased ABA sensitivity to exogenous ABA and proline accumulation, finally enhancing salt and drought tolerance.

As a central regulator of abiotic stress responses, endogenous ABA can participate in the regulation of a variety of physiological responses of plants to abiotic stresses, such as drought, salt, and chilling stress, and regulates the expression of ABA-dependent abiotic stress tolerance-responsive genes (Zhu, 2002; Sripinyowanich et al., 2013; Zhai et al., 2016; Li et al., 2017; Li G. et al., 2020). The ABA- dependent stress-responsive genes, such as *OsRAB16C*, *OsRAB16D*, *OsRAB21*, and *OsLEA3*, are well-known for their involvement in response to salt and drought stresses of rice (Redillas et al., 2012; Jiang et al., 2016; Li et al., 2017, 2018, 2019). The expression of *OsIAA20* was induced by ABA treatment in rice (Song et al., 2009). The study of Zhang et al. (2021) revealed that the overexpression of *OsIAA20* increased salt and drought tolerance of rice plants through an ABA pathway. The *OsIAA18* gene was involved in plant responses mediated by ABA signaling transduction (Song et al., 2009). In *Arabidopsis*, the overexpression of *OsIAA18* led to an increased ABA content, upregulation of ABA biosynthesis genes, such as *AtZEP*, *AtNCED*, *AtABA2*, and *AtAAO*, and enhanced the action of key rate-limiting NCED enzyme under salt and osmotic tolerance (Li G. et al., 2020). In the present study, the sensitivities of exogenous ABA treatment could be induced in the seedlings by the overexpression of *OsIAA18*, indicating that *OsIAA18* might be a positive regulator of ABA signaling in rice (Figure 5). The enhanced activity of NCED and the upregulated expression of *OsNCED4* and *OsNCED5* and a significant increase of endogenous ABA content were observed in the overexpressing *OsIAA18* and WT plants under salt and drought stresses (Figure 6). Further observation showed that the transcript levels of ABA responsive signaling pathway genes, such as *OsRAB16C*, *OsRAB16D*, *OsRAB21*, and *OsLEA3* were markedly upregulated in the overexpressing *OsIAA18* lines (Figure 6). The differences in the salt and drought tolerance between overexpressing *OsIAA18* and WT lines might be, at least in part, due to the reinforced expression of *OsRAB16C*, *OsRAB16D*, *OsRAB21*, and *OsLEA3* and possibly other contributing stress-responsive genes in the overexpressing *OsIAA18* lines. The results suggest that the enhanced salt and drought tolerance exhibited by *OsIAA18* overexpression might be conferred by the coordinated work of these downstream functional genes. Thus, it is suggested that the overexpression of *OsIAA18* enhances the tolerance to salt and drought stresses due to the upregulation of genes involved in ABA biosynthesis, leading to the increased production of ABA, as a signaling molecule to further regulate the expression of ABA-responsive genes (Figure 9).

### *OsIAA18* Increases Salt and Drought Tolerance by Activating Proline Biosynthesis Pathway in Rice

Proline accumulation can enhance salt and drought tolerance in many plant species (Krasensky and Jonak, 2012; Zhang et al., 2019). ABA, salt, and drought treatments induced the expression of *OsP5CS* and the conferred stress tolerance was resulting from an upregulated expression of *OsP5CS* which increased the proline content in rice plants (Xiang et al., 2007; Sripinyowanich et al., 2013; Jiang et al., 2016; Li et al., 2018). P5CS, a catalyzing proline biosynthesis, is critical for enhanced salt and drought tolerance (Jiang et al., 2016). The upregulation of *P5CS* has been shown to increase proline content, resulting in enhanced salt and drought tolerance (Krasensky and Jonak, 2012; Liu et al., 2014; Jiang et al., 2016; Zhai et al., 2016; Kang et al., 2018). As shown in Figure 7, the overexpressing *OsIAA18* lines exhibited the upregulated expression levels of *OsP5CS1* and *OsP5CS2* and increased the content of proline under salt and drought stresses. More proline accumulation in the overexpressing *OsIAA18* rice lines might maintain the osmotic balance between the intracellular and extracellular environment and protect membrane integrity, resulting in enhanced salt and drought tolerance (Liu et al., 2014; Wang et al., 2016; Zhai et al., 2016; Zhang et al., 2019; Li W. et al., 2020). It is suggested that the overexpression of *OsIAA18* enhances the tolerance to salt and drought stresses due to the upregulation of proline biosynthesis gene *OsP5CS*, which increases the production of proline, leading to enhanced salt and drought tolerance (Figure 9).

### *OsIAA18* Improves Salt and Drought Tolerance by Reducing Reactive Oxygen Species Accumulation in Rice

Under abiotic stresses, the increased production of ROS leads to oxidative stress in plants (Hussain et al., 2019; Zhu et al., 2020). Higher ROS levels induce lipid peroxidation in plants and cause injury to cell membranes (Zhao et al., 2018; Hussain et al., 2019). MDA, as a reflection of cellular membrane degradation or dysfunction, is an important intermediary agent in ROS-scavenging (Zhu et al., 2020). It is important to maintain a stronger ROS-scavenging ability under salt and drought stresses to alleviate the induced oxidative damage, especially in the plant leaves where photosynthesis is dramatically impacted (Gill and Tuteja, 2010). The higher ability of ROS-scavenging enzymes decreased the overaccumulated ROS levels, leading to enhanced salt and drought tolerance (Apel and Hirt, 2004; Jiang et al., 2016). SOD and POD play an important role in scavenging ROS by detoxifying H_2_O_2_ and O_2_^–^ into water and stable oxygen, leading to enhanced stress tolerance (Wang et al., 2018; Zhu et al., 2020). In this study, the overexpressing *OsIAA18* rice lines exhibited lower levels of DAB and NBT staining and H_2_O_2_ and MDA content under salt and drought stresses (Figure 8). This indicates a putative role of *OsIAA18* in ROS-scavenging and salt and drought tolerance. We also found that the systematic upregulation of ROS scavenging genes (*OsCu/Zn-SOD1* and *OsPOD8.1*) and significant increase of antioxidant enzyme (SOD and POD) activities were observed in the overexpressing *OsIAA18* rice lines under salt and drought stresses (Figure 8). Therefore, the improved salt and drought tolerance of the transgenic rice plants might be, at least in part, due to the enhanced ROS scavenging capacity (Liu et al., 2014; Zhai et al., 2016; Wang et al., 2018; Li W. et al., 2020). It has been reported that proline is an effective scavenger of singlet oxygen and hydroxyl radicals (Liu et al., 2014; Zhai et al., 2016; Zhang et al., 2019). Our results support that more proline accumulation activates ROS scavenging system, leading to enhanced salt and osmotic tolerance in the overexpressing *OsIAA18* rice lines (Zhai et al., 2016; Wang et al., 2018; Li W. et al., 2020; Figure 9).

## Conclusion

In this study, the overexpression of *OsIAA18* significantly enhanced tolerance to salt and drought stresses in transgenic rice plants. Our results suggest that *OsIAA18* gene may play an important role in regulating salt and drought tolerance by regulating stress-induced ABA signaling. The *OsIAA18* gene is a hopeful candidate for enhancing tolerance to abiotic stresses in plants.

## Data Availability Statement

The datasets presented in this study can be found in online repositories. The names of the repository/repositories and accession number(s) can be found in the article/Supplementary Material.

## Author Contributions

FW and HN conceived and designed the experiments. FW, HN, YL, DX, GL, FZ, and MQ performed the experiments. FW, HN, YL, DX, GW, ZL, GL, FZ, MQ, YY, ZW, BP, LH, CY, and XC analyzed the data. FW, HN, and YL wrote the paper. All authors contributed to the article and approved the submitted version.

## Conflict of Interest

The authors declare that the research was conducted in the absence of any commercial or financial relationships that could be construed as a potential conflict of interest.

## Publisher’s Note

All claims expressed in this article are solely those of the authors and do not necessarily represent those of their affiliated organizations, or those of the publisher, the editors and the reviewers. Any product that may be evaluated in this article, or claim that may be made by its manufacturer, is not guaranteed or endorsed by the publisher.
