# Supplementary material for: OsIAA18, an Aux/IAA Transcription Factor Gene, Is Involved in Salt and Drought Tolerance in Rice
Source: Front Plant Sci. 2021 Nov 18;12:738660. doi: 10.3389/fpls.2021.738660 (PMC8637529; doi:10.3389/fpls.2021.738660)
Supplement: Supplementary file 1 [file Data_Sheet_1.docx]

**
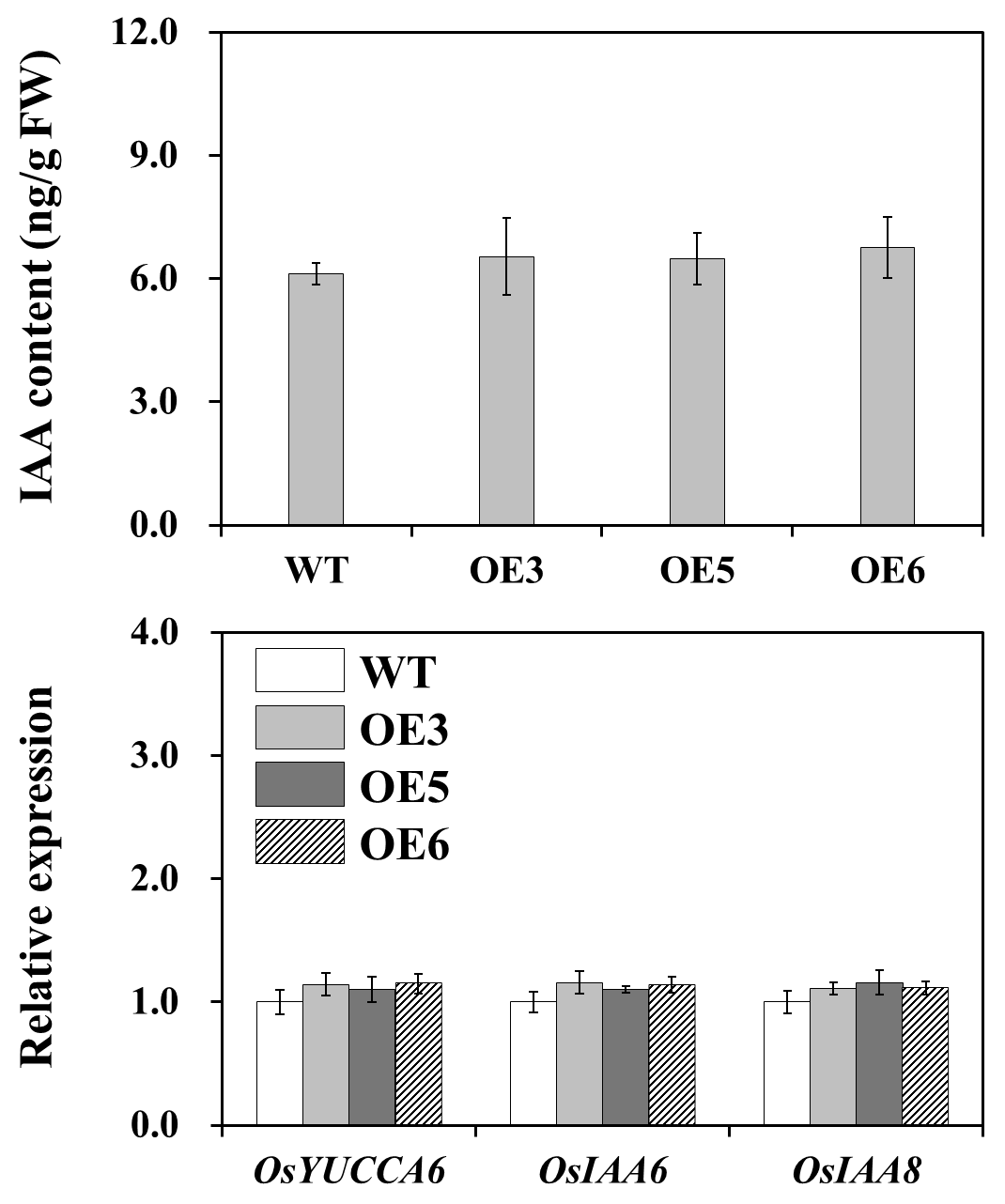
**

**FIGURE S1│IAA content and the expression of IAA signaling related genes in the leaves of *OsIAA18* overexpression and WT plants under control growth conditions**. Data are presented as means ± SE (n=3). * and ** indicate a significant difference from that of WT at *P* < 0.05 and < 0.01, respectively, by Student’s *t*-test.

**Table S1│Primers used in this study.**

| Primer name | Primer sequence (5′-3′) |
| --- | --- |
| Primers for identifying transformants | |
| *hpt*Ⅱ-PCR-F | ACAGCGTCTCCGACCTGATGCA |
| *hpt*Ⅱ-PCR-R | AGTCAATGACCGCTGTTATGCG |
| Primers for real-time quantitative PCR | |
| *OsIAA18*-F | AAGAATGTGGGAAGGAGCTAACG |
| *OsIAA18*-R | ATGGTGGTGAGGGACAGCAT |
| *OsYUCCA6*-F | CCATTCCCAGATGGTTGGAAGG |
| *OsYUCCA6*-R | CATGTTGCGCCTCAAGATATTTG |
| *OsIAA6*-F | GGCTATCGTCAGCTGTCAAACA |
| *OsIAA6*-R | GCAATTTGCGCATTAGTTTGG |
| *OsIAA8*-F | CCGCTAGACGGCTACAAAGG |
| *OsIAA8*-R | GGTGATGGATGCTCTGAACATG |
| *OsNCED4*-F | GATTGCACGGCACCTTCATT |
| *OsNCED4*-R | CTCTGTAATTTGATTTTTCACTGGCTAAT |
| *OsNCED5*-F | GGATGGGCTGAACTTCTTCCAG |
| *OsNCED5*-R | CAGCACATTCGTGATGAACCCT |
| *OsRAB16C-*F | TTCCCGGCCAGCACTAAAT |
| *OsRAB16C-*R | AAACTGCACGTACATCACGACAT |
| *OsRAB16D-*F | CGGGTAAACAATAAAGTCGTGATG |
| *OsRAB16D-*R | GCGCACTTACATACAGTGCTACGT |
| *OsRAB21-*F | CACACCACAGCAAGAGCTAAGTG |
| *OsRAB21-*R | TGGTGCTCCATCCTGCTTAAG |
| *OsLEA3-*F | GCCGTGAATGATTTCCCTTTG |
| *OsLEA3-*R | CACACCCGTCAGAAATCCTCC |
| *OsP5CS1-*F | CCCGTCCCGGAGCTTCGTGAG |
| *OsP5CS1-*R | CCTAAGTCGCTGTCGCCCCAC |
| *OsP5CS2-*F | GCTGCCGTCGGTCAGAGTG |
| *OsP5CS2-*R | CTCGTATGGTTGCCTCCTGGT |
| *OsCu/Zn-SOD1-*F | TCAATCATTGGCAGAGCCGT |
| *OsCu/Zn-SOD1-*R | ACAGCCAGATCCCCCTTACT |
| *OsPOD8.1-*F | CTGCTCCAAAGTGAACTAATAATTAAGTAAAG |
| *OsPOD8.1-*R | CCCAGCCTTATTCCCAAATTT |
| *Osactin*-F | TTATGGTTGGGATGGGACA |
| *Osactin*-R | AGCACGGCTTGAATAGCG |
